# Characteristics and Functional Properties of Maillard Reaction Products from α-Lactalbumin and Polydextrose

**DOI:** 10.3390/foods12152866

**Published:** 2023-07-27

**Authors:** Kexin Dai, Jiangpeng Wang, Yingting Luo, Yaqi Tu, Fazheng Ren, Hao Zhang

**Affiliations:** 1 College of Food Science and Nutritional Engineering, China Agricultural University, Beijing 100083, China; 2019309080302@cau.edu.cn (K.D.); 2019306100717@edu.cau.cn (J.W.); s20213061018@cau.edu.cn (Y.L.); s20193060941@cau.edu.cn (Y.T.); 2 Beijing Laboratory of Food Quality and Safety, Department of Nutrition and Health, China Agricultural University, Beijing 100091, China; renfazheng@cau.edu.cn; 3 Food Laboratory of Zhongyuan, Luohe 462300, China

**Keywords:** polydextrose, α-lactalbumin, Maillard reaction, hydrophobicity, antioxidant activity

## Abstract

The characteristics and the functions of Maillard reaction products (MRPs) produced by polydextrose (PD), a new type of prebiotic, and α-lactalbumin (α-LA) were valued. PD and α-LA were incubated at 60 °C and 79% relative humidity for up to 72 h to prepare MRPs. The results showed that the absorbance and fluorescence intensity of heated α-LA-PD increased, and the amount of free amino groups reduced as the reaction progressed, which confirmed the formation of different stages of MRPs. Electrophoresis revealed an increase in molecular mass and the degree of covalent cross-linking. The secondary structure of MRPs experienced no significant changes with the measurement of circular dichroism (CD), while the tertiary structure gradually unfolded, exposing hydrophobic groups. Furthermore, a significant increase was detected in the radical-scavenging activity of 2,2-diphenyl-1-picrylhydrazyl (DPPH) and the ferric reducing/antioxidant power (FRAP) of MRPs. The findings offer a foundation for understanding the structural and functional features of MRPs in formula milk powder.

## 1. Introduction

The Maillard reaction is the condensation reaction that occurs between carbonyl and amino groups [1]. It consists of a complexity of reactions, not a singular one, for which the progress and consequences are greatly influenced by variables including pH, time, and temperature. As a result, heating at a relatively low temperature gradually produces the same color outcomes as roasting at a higher temperature in a shorter time as long as the moisture of the environment is not too low [2]. For example, during cookie baking or bread toasting, with similar moisture content in the dough, the same brown color can be achieved at a higher temperature for a short period and at a lower temperature for a longer period. The Maillard reaction can be separated into the following reaction phases by analyzing the reaction mechanism: The prior stage is characterized by no color and ultraviolet (UV) absorption with glycosamine condensation and Amadori rearrangement. The degradation and fragmentation of carbohydrates as well as the degradation of amino acids take place in the intermediate stage. The final phase is represented by browning, aldehyde-amine condensation, and aldol condensation [3]. The Maillard reaction can not only change the color and aroma of food but also impacts bioactivities, such as antioxidant activity, hydrophobicity and antigenicity [3]. Infant formulas are rich in protein and carbohydrates, which provide ample raw materials for the Maillard reaction. The content of Maillard reaction products (MRPs) is almost zero in raw milk, but the content increases with a variety of thermal processes utilized in manufacturing and shelf life storage at room temperature [4]. Heat-induced protein structural changes, such as denaturation, aggregation, cross-linking, and undesirable thermal breakdown products, decrease the protein’s functional properties and absorption [5]. Since infant formulas are frequently the only nutritional source during a substantial period of rapid infant development and growth, it is critical to produce secure and nutritionally appropriate items that meet newborns’ needs. This reinforces the need for further study of the Maillard reaction.

Among all of the regularly supplemented proteins in infant formulas, α-lactalbumin (α-LA) is the second-most prevalent protein in bovine whey as well as a plentiful protein in human breastfeeding. With a total molecular mass of 14,186 Da, α-LA contains 123 amino acid residues which include eight cysteines covalently linked through four disulfide bonds. Not only necessary amino acids such as lysine, phenylalanine, and tryptophan are provided by α-LA to nourish infants, but α-LA promotes mineral binding and releases bioactive peptides with a wide range of functions as digested, as well as promoting protein synthesis in developing newborns [6]. α-LA, derived from bovine sources, is an ideal replacement due to its high sequence similarity with α-LA from humans. Since α-LA, as one of the most abundant proteins in bovine milk, has a significant number of lysine amino acid residues in the primary structure, it is more prone to participate in the Maillard reaction than other milk proteins, because of the amino group present in the side chain of lysine [7].

Various nutritional carbohydrates, such as monosaccharides, prebiotic oligosaccharides, and polysaccharides, are also present in infant formulas. One of them is polydextrose (PD), a synthetic polymer of glucose, which is created via vacuum polycondensation at a high temperature from a mixture composed of glucose, citric acid, and sorbitol in a certain ratio. PD is a D-glucose polymer with a 1,6-glycosidic bond as the mainstay. PD has a variety of advantageous properties in food processing, including high water solubility (80% *w*/*w* at 25 °C), high stability and dispersibility, and low sweetness, but is sufficient in mouthfeel. As a widely acknowledged prebiotic, PD is not able to be digested and absorbed by the small intestine, and most of it is excreted with feces, similar to dietary fiber. PD is conducive to the proliferation of beneficial intestinal bacteria, promotes the generation of short-chain fatty acids and inhibits intestinal spoilage bacteria that can effectively regulate the balance of intestinal flora and promote the absorption of nutrients such as calcium [8]. Because of its rich physiological functions, polydextrose receives a wide range of attention in various research fields, such as food and health care.

The Maillard reaction between several commonly seen reducing sugars and milk proteins in infant formula has already been investigated, e.g., the reaction between lactose and casein or whey proteins [9]. Glucose, maltose, galactose and fructose have been reported to react with α-LA, which makes a difference in biological activities, e.g., antioxidant activity, antigenicity, hydrophobic activity, digestibility of MRPs, and emulsion stability have been improved [2,10,11]. The characteristics and functions of MRPs were different among different sugars. To be specific, compared to glucose-based MRPs, the degree of browning in galactose examples was obviously higher [10]. MRPs of glucose and α-LA showed a significantly higher antioxidative capacity and surface hydrophobicity than those of fructose and α-LA, which may modify the functionality and digestibility of products [2]. Among all these biological activities, the reason why antioxidant activity is widely measured in dairy products is because this type of endogenous antioxidant not only improves product storage stability but also increases the antioxidant capacity of the gastrointestinal tract as reported [3]. However, as a novel prebiotic substance added to infant formula, the structural characterizations and functions of MRPs obtained from α-LA and PD remain unknown.

Therefore, in this study, we focused on the characteristics and functions of MRPs formed by α-LA and PD. Absorbance and fluorescence intensity were valued to show the MRPs from different stages of formation. In order to estimate the level of reaction, the content of free amino groups was counted. SDS-PAGE (Sodium dodecyl sulfate polyacrylamide gel electrophoresis) was applied to determine the molecular mass of products. The secondary structure of MRPs and the tryptophan group condition were determined by utilizing circular dichroism (CD) and fluorescence spectral scanning, respectively. As for the functions of products, the exposure of hydrophobic groups was assayed. The radical-scavenging activity of 2,2-diphenyl-1-picrylhydrazyl (DPPH) and ferric reducing/antioxidant power (FRAP) were employed as indicators to evaluate the antioxidant properties of MRPs.

## 2. Materials and Methods

### 2.1. Chemicals

α-LA (L6010, purity ≥ 85%, biological source: bovine milk), 8-anilino-1-naphtha-lenesulfonic acid ammonium salt (ANS), 2,2-diphenyl-1-picrylhydrazyl (DPPH) and 2,4,6-tris(2-pyridyl)-striazide (TPTZ) were provided by Sigma-Aldrich Chemical Co. (St. Louis, MO, USA). PD (purity ≥ 90%, 80–120 mush, average degree: 13 (range from 2 to 120), average molecular weight: 3200 (162–5000 molecular weight: 88.7%), provided by manufacturer) was purchased from Shandong Bailong Chuangyuan Biotechnology Co., Ltd., (Dezhou, China). Sodium tetraborate decahydrate, ophthal-aldehyde (OPA) and sodium dodecyl sulfate (SDS) were all obtained from Aladdin Reagent (Shanghai, China). β-Mercapto-ethanol was acquired from Kulaibo Technology Co., Ltd., (Beijing, China). All additional chemicals were of an analytical grade, provided by Yongda Chemical Reagent Co., Ltd., (Tianjin, China).

### 2.2. Preparation of α-LA-PD MRPs

α-LA was weighed and dissolved in ultra-pure water to 50 mg/mL and PD weighed in the same way as α-LA was added (mass ratio of α-LA: PD was 1:1). After lyophilization, the mixture was incubated at 60 °C for 0, 12, 24, 48 and 72 h under 79% relative humidity (RH), formed by saturated potassium bromide solution [12]. The mixture for each time point was prepared separately in triplicate before lyophilizing. α-LA incubated without PD, and PD incubated alone were regarded as control groups. The protein concentration of various samples after incubation was determined utilizing BCA assay [13]. The samples are divided into vials according to the requirement of each experimental indicator and maintained at −20 °C until measuring. The solution utilized for sample dilution is ultra-pure water. The preparation methodology of α-LA-PD MRPs simulates the process of milk powder processing. The setting of temperature, time and humidity in this method was used to speed-up the Maillard reaction, which simulated the interaction between protein and saccharides in milk powder during shelf life [12].

### 2.3. Measurement of Absorbance and Fluorescence Intensity

Samples were diluted to 1 mg/mL protein concentration with ultra-pure water for 294 nm absorbance measurement, and 16 mg/mL protein concentration for 420 nm absorbance determination using a spectrophotometer, respectively [14]. The ultra-pure water was regarded as a blank control.

Fluorescence intensity was monitored according to the methods of Jing and Kitts [15]. After dissolving the sample in ultra-pure water to a concentration of 0.5 mg/mL, the intensity of fluorescence was measured at 420 nm after excitation at 340 nm. Ultra-pure water was used as a blank control.

### 2.4. Measurement of Free Amino Groups

The OPA assay was applied to determine the amount of free amino groups [16]. OPA reagent was prepared by dissolving 40 mg of OPA in 1 mL of anhydrous ethanol, and then 25 mL of 0.1 M sodium tetraborate, 100 μL of β-mercapto-ethanol, and 2.5 mL of 20% (*w*/*v*) SDS were added. In the last step, ultra-pure water was added up to 50 mL. After diluting the sample to 6 mg/mL of protein concentration, a 20 μL sample was mixed well with 200 μL of OPA reagent. After incubation for 2 min at room temperature, the intensity of absorbance at 340 nm was measured against ultra-pure water as a blank control. A glycine solution was used for the preparation of the calibration curve (0–20 mM). The decrease in the content of free amino groups in the incubation mixture was shown by a relative concentration (%) compared with the content of free amino groups at 0 h which, was regarded as 100% content of amino groups available for the Maillard reaction.

### 2.5. SDS-PAGE

A 12% acrylamide separating gel (pH 8.8) and a 6% acrylamide stacking gel (pH 6.8) were utilized to perform SDS-PAGE after a modification of the method used by Laemmli et al. [17]. Samples were prepared with the addition of β-mercapto-ethanol and a protein concentration of 2 mg/mL. Gels were dyed with 0.5% Coomassie Brilliant Blue R-250 for 1 h following electrophoresis, then removed from staining until distinct bands could be seen, before being photographed.

### 2.6. CD and Tryptophan Fluorescence Spectroscopy

The secondary structures of α-LA were determined using a CD spectrophotometer (J-810, JASCO Corporation, Tokyo, Japan). Samples were diluted to 0.2 mg/mL of protein concentration with ultrapure water. At a scan rate of 100 nm/min with a 1 nm bandwidth, spectra ranging from 190 nm to 260 nm were recorded in 1 mm quartz cuvettes. Circular Dichroism Spectroscopy Deconvolution Version 2.0d (CDNN) was used to calculate the secondary structure after removing background values.

A spectrofluorometer (J-810, JASCO Corporation, Japan) was used for the measurement of tryptophan fluorescence. Prior to the measurement, samples were diluted to 1 mg/mL of protein concentration with ultra-pure water. The emission spectrum of tryptophan was recorded from 300 to 400 nm with a slit width of 3 nm, after excitation at 280 nm [18].

### 2.7. Surface Hydrophobicity (H_0_)

The H_0_ was identified using a type of fluorescence probe, 8-anilino-1-naphthalene sulfonic acid (ANS), which has an extremely high affinity for the hydrophobic region of proteins and can bind to the low polarity hydrophobic surface of proteins, on the basis of the method of Zhong et al. [19]. α-LA and MRPs were respectively diluted to 0.0125–0.2 mg/mL (0.0125, 0.025, 0.05, 0.1, 0.2) using 0.02 mM phosphate buffer (pH 7.0). The same buffer was also used to dilute the ANS solution (8.0 mM). An 800 µL sample was mixed with 4 µL ANS solution. After 15 min of darkness reaction, the fluorescence intensity was determined at 470 nm (emission) and 370 nm (excitation). Making a plot with the protein concentration (0.0125, 0.025, 0.05, 0.1, 0.2 mg/mL) as the abscissa and the fluorescence intensity as the ordinate, the initial slope of the linear regression regarding H_0_ was calculated.

### 2.8. Measurement of the Antioxidant Activity

#### 2.8.1. DPPH Radical-Scavenging Activity

The radical-scavenging activity of 2,2-diphenyl-1-picrylhydrazyl (DPPH) was determined based on the previous method of Tu et al. [20], in which 40 µL protein solution (10 mg/mL protein concentration) was well mixed with 200 µL DPPH solution (0.25 mM), prepared with ethanol on the day. Then, the mixture was left covered for 30 min at room temperature before centrifuging it at 6000× *g* for 2 min (4 °C). After collecting the supernatant, its absorbance at 517 nm was measured. The blank replaced samples with equivalent ultra-pure water. Antioxidant activity calculation method was as follows:

DPPH radical scavenging activity (%) = (1 − A_517_ sample/A_517_ blank) × 100% (1)

#### 2.8.2. Ferric Reducing Antioxidant Power (FRAP)

The FRAP of MRPs was assayed based on Benzie & Strain [21]. After adding a 30 µL sample (10 mg/mL protein concentration) to a 150 µL FRAP solution (pH 3.6) containing TPTZ (10 mM, 1 mL, dissolved in a 40 mM concentrated hydrochloric acid), FeCl_3_ (20 mM, 1 mL, dissolved in ultra-pure water) and acetate buffer (300 mM, 10 mL, prepared with ultra-pure water), the absorbance of the mixture was assessed at 595 nm after being heated at 37 °C for 30 min. MRPs were replaced by α-LA in the control group. The equivalents of ascorbic acid (0–1000 µM) were used to evaluate antioxidant activity.

### 2.9. Statistical Analysis

Data were analyzed using IBM SPSS Statistics 25 and each experiment was carried out in triplicate. One-way ANOVA (Analysis of Variance) was used to construct the statistical analysis relying on the Tukey test. Statistical differences were deemed significant as *p* < 0.05.

## 3. Results and Discussion

### 3.1. Absorbance and Fluorescence Intensity

**Figure 1:**
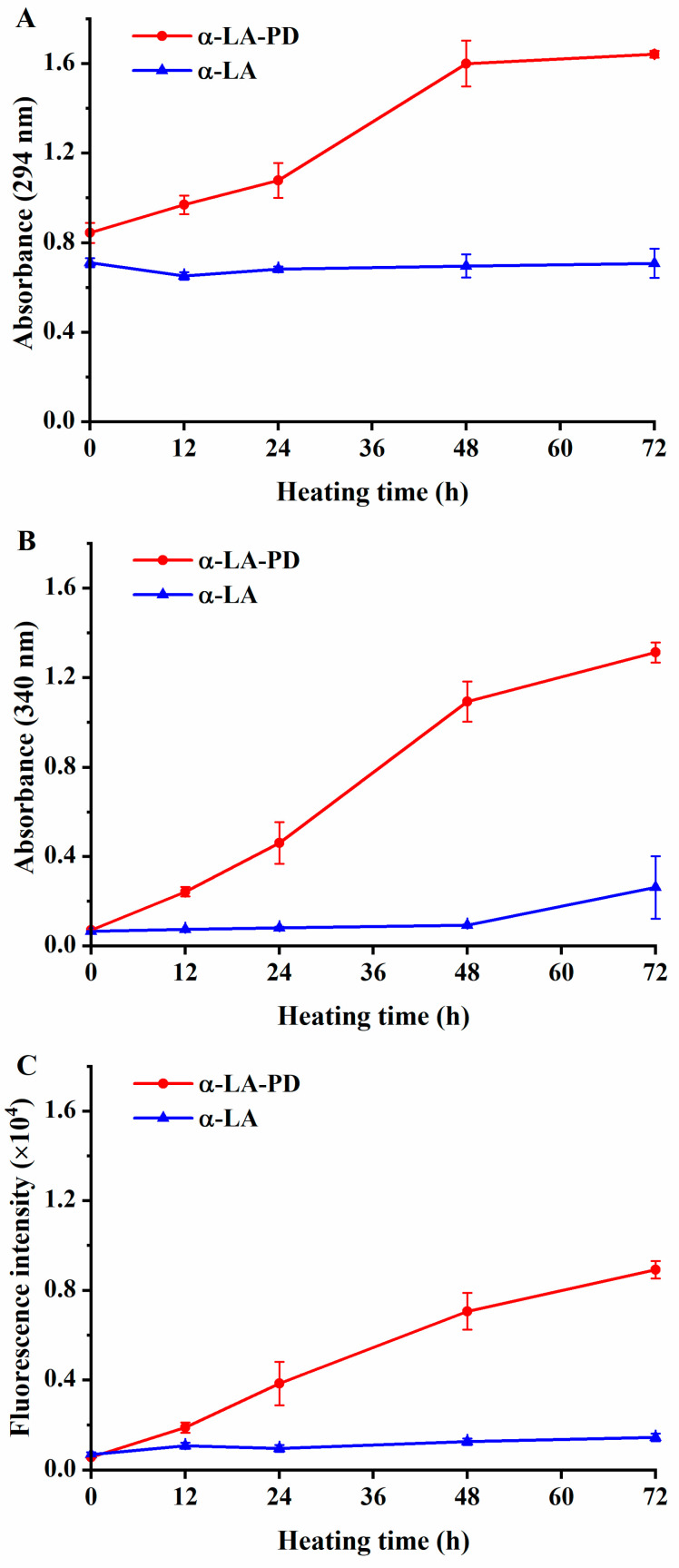
Absorbance at 294 nm (**A**), absorbance at 420 nm (**B**) and fluorescence intensity at 350/420 nm (excitation/emission) (**C**) of α-LA-PD and α-LA during heating 72 h at 60 °C and 79% RH. Data are presented as mean ± SD of the triplicate experiment.

As shown in Figure 1A,B, no significant difference (*p* > 0.05) was seen between A_294_ and A_420_ when α-LA was heated without PD. Additionally, changes in A_294_ and A_420_ when PD was heated alone were negligible. In contrast, the absorbance of α-LA-PD increased significantly (*p* < 0.05). Because the color of the MRP starts to change with the appearance of UV absorption in the middle stage and becomes intensely colored in the final process, 294 nm and 420 nm absorption were measured to confirm the progress of the Maillard reaction at each stage. Increase in absorbance indicates that the intermediate products were formed between α-LA and PD and then later melanoidins were developed [9].

Changes in fluorescence intensity were negligible when α-LA was heated without PD (Figure 1C), and when PD was heated without α-LA, the result was the same. The formation of fluorescent compounds is considered as an association with the Maillard reaction [22]. Fluorescence intensity significantly (*p* < 0.05) increases during the heating of the mixture of α-LA and PD. The development of fluorescence could be explained by the Strecker degradation of amines, as well as reactions, which occur between intermediate substances and reductive products [19]. As a result, changes in fluorescence intensity are always applied as the precursors of brown products, revealing the different period of Maillard reaction with absorbance [23].

### 3.2. Variations in Free Amino Group Contents

**Figure 2:**
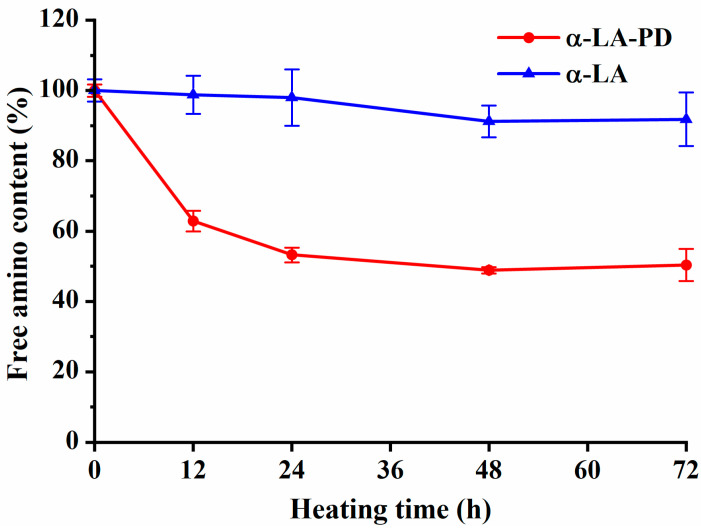
Free amino group content of α-LA-PD and α-LA during heating 72 h at 60 °C and 79% RH. Data are presented as mean ± SD of the triplicate experiment.

The OPA method was used to evaluate the change in amino group content during Maillard reaction between α-LA and PD. During the absence of PD, no significant difference (*p* > 0.05) was observed (Figure 2). In contrast, in the mixture α-LA and PD, approximately 50% free amino groups were lost after heating for 24 h, which indicates that the Maillard reaction was occurring between them [24]. Further heating of the mixture up to 72 h did not significantly lead to a decrease in free amino group content. This indicated that heating quickly induced the reaction of α-NH2 and ε-NH2 covalently attached to α-LA with the carbonyl group of PD, which leads to the depletion of free amino groups [17]. The reduction of OPA confirmed the occurrence of the Maillard reaction, together with the increase of absorbance and fluorescence intensity.

### 3.3. SDS-PAGE

**Figure 3:**
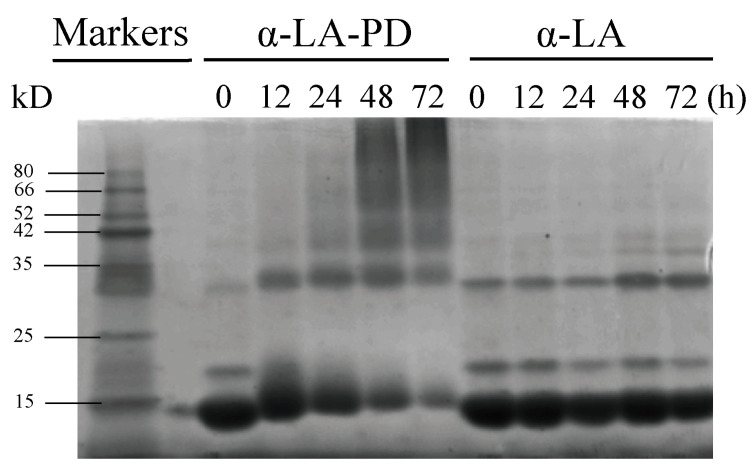
SDS-PAGE reducing gels of heated samples at 60 °C and 79% RH. Bands from left to right are markers, α-LA-PD and α-LA heated at 0, 12, 24, 48 and 72 h.

Analysis of the molar mass distribution of MRPs produced by α-LA and PD was carried out by SDS-PAGE (Figure 3). The unheated samples showed a strong band at 14 kDa and 28 kDa, which represented the monomers and dimers of α-LA. When α-LA was heated alone, the dimer band deepened at 48 and 72 h. The trimer bands (around 42 kDa) were also observed with prolonged heating. As a result, heating promoted the formation of the dimer and trimer structure of α-LA. For MRPs, as heating proceeded, the α-LA bands moved towards higher molecular weight, illustrating the conjugation of α-LA with PD. In addition, the appearance of high molecular weight bands (>35 kDa) during heating indicated that cross-linking between protein molecules also occurred [25]. The 18 kDa band that appeared in the α-LA control group corresponded to β-lactoglobulin, because the purity of α-LA is not 100%. The Maillard reaction between β-lactoglobulin and PD resulted in the 18 kDa band becoming shallow more quickly than the α-LA band and disappearing [26]. Since the α-LA we used was from multiple batches, the difference in intensity of ~30 kDa between the two 0 h samples is due to the slight difference in β-casein content between batches of α-LA product [27]. Since the overall proportion of α-LA is above 85%, the existence of other proteins would not affect the leading results of the experiment. Not only this, but with the improvement of molecule size during protein conjugation, a larger surface area would form at the water-in-oil interface, resulting in higher amphiphilic activity, which reached an agreement with the hydrophobicity analysis discussed below [28].

### 3.4. CD and Tryptophan Fluorescence Spectroscopy

**Figure 4:**
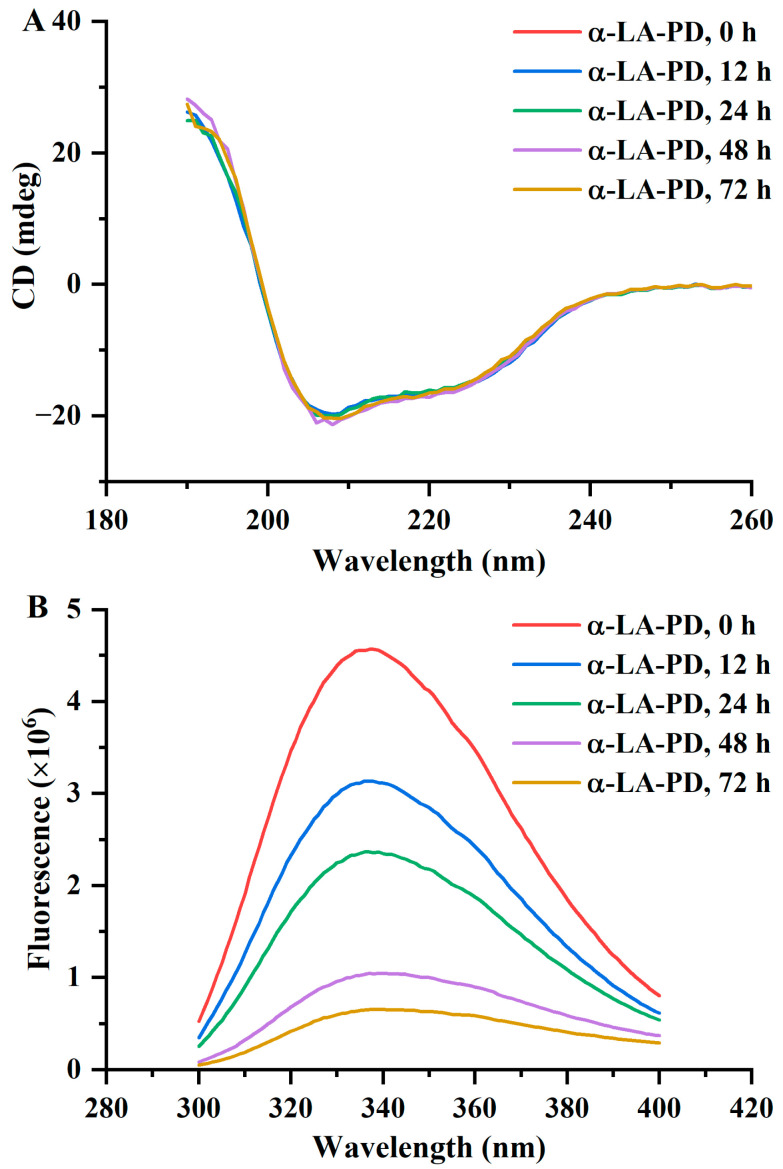
CD (**A**) and fluorescence spectra at 280 nm excitation (**B**) of α-LA-PD and α-LA during heating for 72 h at 60 °C and 79% RH. Data are presented as mean ± SD of the triplicate experiment.

CD was used to determine the difference in absorption of left- and right-handed circularly polarized light [18]. The main chromophore of CD in the far-ultraviolet band of 190–260 nm was the peptide chain. Differences in absorption of left- and right-handed circularly polarized light by protein can be used for evaluation of changes of its secondary and tertiary structure. The peptide bond is the main chromophore which absorbs polarized light in the far-ultraviolet region (190–260 nm) in CD spectroscopy of proteins. Therefore, differences in absorbance of proteins in this region of CD spectroscopy can be used for the evaluation of changes in their secondary structure [29]. In the non-heated sample, two intensely negative bands at 208 nm and 222 nm were observed (Figure 4A), indicating that α-LA has a high α-helical protein content. CD spectra of samples heated for different time showed similar results. The percentages of α-helix, β-sheet, β-turn and random coil for non-heated samples were 31.6%, 21.7%, 18.0% and 28.7%, respectively (Table 1). There was no notable change (*p* > 0.05) from the secondary structure data in α-LA-PD with heating time. According to Tu et al. (2020), the conjugates of α-LA with dextran revealed an improvement in β-sheet and a loss in α-helix simultaneously but the conjugates of α-LA with 2′-fucosyllactose did not change significantly [20]. Therefore, variations in the secondary structure of α-LA might be associated with the type of carbohydrates participating in the Maillard reaction. With the production of macromolecules and the cross-link indicated in SDS-PAGE, the secondary structure of protein did not change significantly but the tertiary structure unfolded, coinciding with the increase in hydrophobicity and decrease in fluorescence intensity.

The change in fluorescence spectra can reflect the polar environment of tryptophan and its interaction with surrounding groups [30]. No significant change (*p* > 0.05) was observed in α-LA after 72 h of heating. In Figure 4B, as the heating time increased, the fluorescence spectra of MRPs decreased significantly (*p* < 0.05). The maximum emission wavelength had a small blueshift trend, as in the results of Boggione Santos et al. [31], suggesting that the protein had maintained its native formation. The lower fluorescence intensity indicated that, as the protein structure unfolded, tryptophan residues, originally wrapped inside the protein, were exposed to a polar environment, which decreased the fluorescence spectra of tryptophan [31].

### 3.5. Surface Hydrophobicity (H_0_)

**Figure 5:**
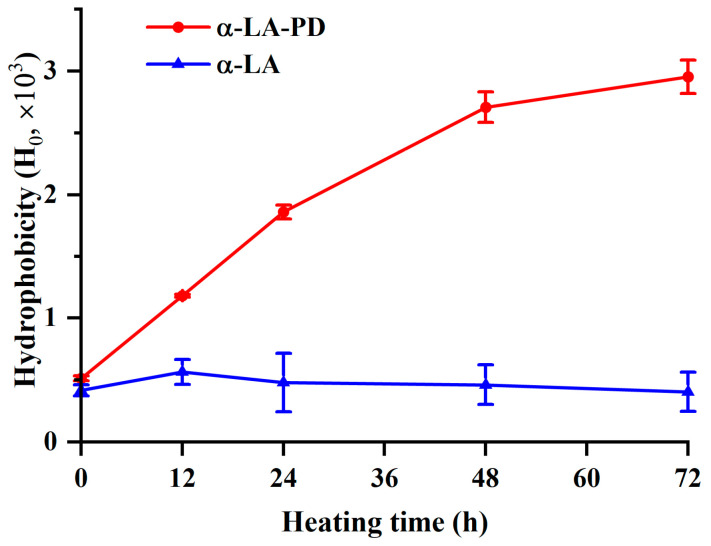
Hydrophobicity of α-LA-PD and α-LA during heating for 72 h at 60 °C and 79% RH. Data are presented as mean ± SD of the triplicate experiment.

Protein surface hydrophobicity is closely related to its functional characteristics [32]. In addition to being the most accurate predictor of a protein’s surface hydrophobicity, ANS also seems to be the simplest experimental technique to use [33]. Fluorescent probe ANS was used to determine H_0_ values (Figure 5). The H_0_ of MRPs significantly increased (*p* < 0.05) after thermal processing, while for the H_0_ of α-LA treated alone no significant difference (*p* > 0.05) was observed. According to Joubran et al. [10], the hydrophobicity of α-LA increased after the Maillard reaction with fructose or fructo-oligosaccharide (FOS), compared to native α-LA. The increase in H_0_ should be explained as a consequence of the denaturation of proteins and the revealing of hydrophobic parts of amino acid to the water environment, which led to the cleavage of non-covalent bonds, such as Van der Waals and hydrogen bonds. [34]. Moreover, the unfolding of the protein tertiary structure upon heating was accompanied by a partial shuffling of disulfide bonds, resulting in the externalization of the hydrophobic region, thereby increasing H_0_ [10]. The result was also confirmed by the unfolding of the tertiary structure and the increase of tryptophan, a hydrophobic amino acid, mentioned above.

### 3.6. Antioxidant Activity

#### 3.6.1. DPPH Radical-Scavenging Activity

**Figure 6:**
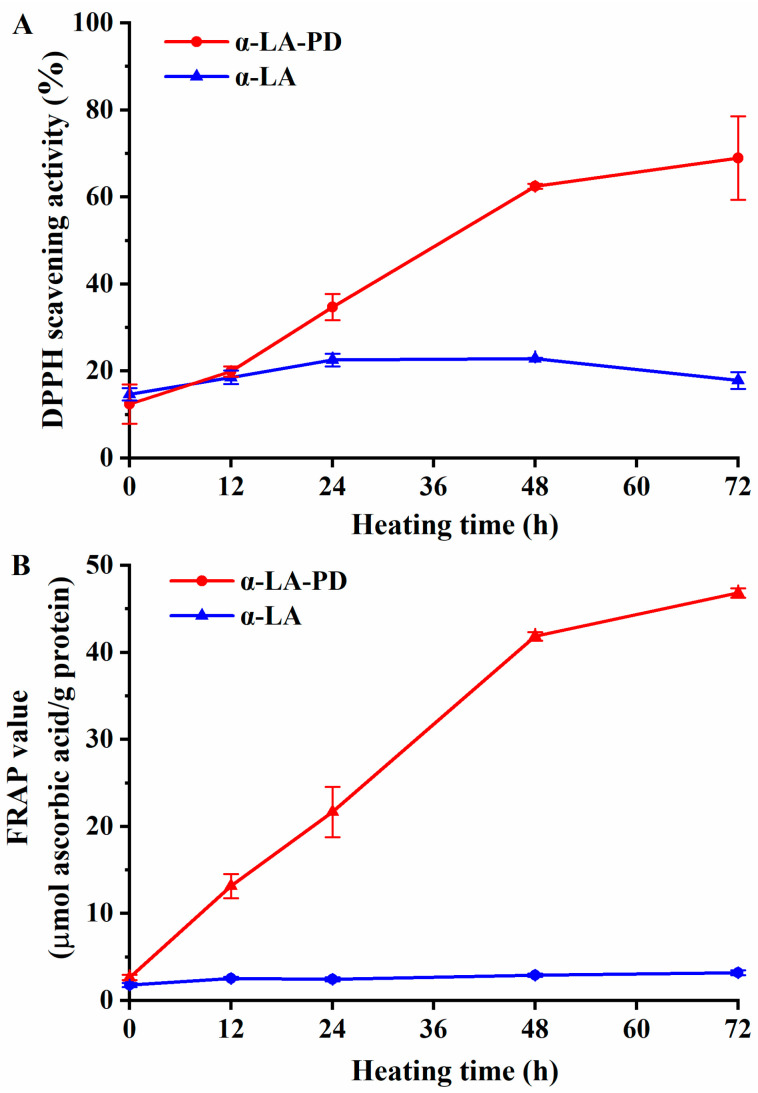
DPPH radical scavenging activity (**A**) and ferric reducing antioxidant power (FRAP) (**B**) of α-LA-PD and α-LA during heating for 72 h at 60 °C and 79% RH. Data are presented as mean ± SD of the triplicate experiment.

DPPH can be reduced by hydrogen atoms transferred from H-donors [35]. In the presence of antioxidants, the single electron of DPPH is trapped and the color becomes lighter, the absorbance at the maximum absorption wavelength declines, and the degree of decrease is linear. Thus, the change of absorbance can reflect the hydrogen-donating capacity of antioxidants. As demonstrated in Figure 6A, the ability of α-LA-PD to scavenge DPPH radicals increased gradually with increasing thermal processing (*p* < 0.05). After heating for 72 h, the ability of DPPH scavenging was significantly improved to 69%. The enhancement of the antioxidant activity of MRPs has been previously reported in some studies. For example, β-lactoglobulin modified with lactose, arabinose, and ribose had, respectively, 10, 60 and 80% of radical scavenging activity [35], which indicates that quantity, the composition of amino acids and the type of sugar and protein can all affect DPPH scavenging ability.

#### 3.6.2. FRAP

FRAP is another assay that is widely utilized to determine the antioxidative capacity of Maillard-type compounds. MRPs’ reducing power is recorded in absorbance units [36]. As shown in Figure 6B, there were no significant differences in FRAP value (*p* > 0.05) of α-LA heated alone. In contrast, the FRAP values of MRPs showed a dramatic rise (*p* < 0.05), consistent with the results from Dong et al. (2012) that MRPs contain a large number of heterocyclic hydrophobic amino acids, which can be regarded as hydrogen donors (or antioxidants) [37]. The Maillard reaction could also produce reducing substances, such as reducing ketones [38], which can increase the antioxidant properties of MRPs. Moreover, there is a similar point as in the DPPH experiment at which the increase in MRPs antioxidant capacity leveled off after 48 h, which means that it is possible to determine the course of the Maillard reaction by variations in antioxidant capacity. As Liu et al. (2014) reported, the formation of large molecular weight compounds was highly consistent with antioxidant capability [39], which contributed to higher stability of the whole MRP system.

## 4. Conclusions

In this study, we reported the characterization and functional properties of MRPs, firstly from α-LA and PD. The reduction in free amino groups content occurred during the Maillard reaction. The cross-linking of α-LA and PD produced macromolecules and brown substances. Interestingly, heating also promotes the formation of dimers and trimers of α-LA. In addition, the tertiary structure of α-LA gradually unfolded, resulting in an increase in the hydrophobicity of the product, while the protein’s secondary structure did not show an obvious change. DPPH radical scavenging activity and FRAP antioxidant capacity increased significantly during the formation of MRPs. Therefore, MRPs could even be used to intervene in some of the redox reactions that occur in the intestine. Interestingly, the Maillard reaction also improves protein stability, which means that we may be able to create a delivery platform to transport proteins or prebiotics deeper into the intestine. However, these treatments modalities require further studies to demonstrate their effectiveness and impact on the gut microbiome. As a potential functional ingredient in milk powder, further work is needed to evaluate the prebiotic properties of α-LA-PD Maillard products.

## Figures and Tables

**Table 1:** The content of α-helix, β-sheet, β-turn and random coil in the secondary structure of α-LA-PD during heating for 72 h at 60 °C and 79% RH.

Samples	α-Helix (%)	β-Sheet (%)	β-Turn (%)	Random Coil (%)
α-LA-PD, 0 h	31.6 ± 0.7	21.7 ± 0.9	18.0 ± 0.1	28.7 ± 0.5
α-LA-PD, 12 h	30.5 ± 1.2	22.4 ± 1.7	18.1 ± 0.2	29.0 ± 0.9
α-LA-PD, 24 h	30.7 ± 0.9	22.5 ± 1.1	18.1 ± 0.2	28.7 ± 0.9
α-LA-PD, 48 h	32.6 ± 0.5	20.9 ± 0.5	18.2 ± 0.1	28.3 ± 0.5
α-LA-PD, 72 h	31.6 ± 0.8	21.7 ± 1.0	18.1 ± 0.2	28.6 ± 0.7

## Data Availability

Data is contained within the article.
